# Chemistry of Nitroquinolones and Synthetic Application to Unnatural 1-Methyl-2-quinolone Derivatives

**DOI:** 10.3390/molecules15085174

**Published:** 2010-07-30

**Authors:** Nagatoshi Nishiwaki

**Affiliations:** School of Environmental Science and Engineering, Kochi University of Technology, Tosayamada, Kami, Kochi 782-8502, Japan; E-Mail: nishiwaki.nagatoshi@kochi-tech.ac.jp; Tel.: +81-887-57-2517; Fax: +81-887-57-2520

**Keywords:** unnatural 1-methyl-2**-**quinolone, *cine*-substitution, regioselective C-C bond formation, nitroalkene, cycloaddition

## Abstract

The 1-methyl-2-quinolone (MeQone) framework is often found in alkaloids and recently attention was drawn to unnatural MeQone derivatives with the aim of finding new biologically active compounds, however, low reactivity of the MeQone framework prevents the syntheses of versatile derivatives. A nitro group is one of the useful activating groups for this framework that enables a concise chemical transformation. Among nitroquinolones, 1-methyl-3,6,8-trinitro-2-quinolone (**TNQ**) exhibits unusual reactivity favoring region-selective *cine*-substitutions that afford 4-substituted 1-methyl-6,8-dinitro-2-quinolones upon treatment with nucleophilic reagents. Contrary to this, 1-methyl-3,6-dinitro-2-quinolone (**3,6-DNQ**) does not undergo any reaction under the same conditions. The unusual reactivity of **TNQ** is caused by steric repulsion between the methyl group at the 1-position and the nitro group at the 8-position, which distorts the MeQone framework. As a result, the pyridone ring of **TNQ** loses aromaticity and acts rather as an activated nitroalkene. Indeed, the pyridone moiety of **TNQ** undergoes cycloaddition with electron-rich alkenes or dienes under mild conditions, whereby a new fused ring is constructed on the [*c*]-face of the MeQone. Consequently, **TNQ** can be used as a new scaffold leading to versatile unnatural MeQone derivatives.

## 1. Introduction

The 1-methylquinolin-2(1*H*)-one (1-methyl-2-quinolone, abbreviated as MeQone hereafter) is one of the fundamental frameworks found in the quinoline alkaloids that are mainly isolated from the Rutaceae family of plants. The MeQone framework is found as a partial structure in more than 300 quinoline alkaloids, and their isolation, structural determination and total syntheses have been energetically pursued in past decades [1,2,3,4,5,6]. While naturally occurring MeQone derivatives are of current research interest because they show a wide range of biological activities such as cytotoxic, antiparasitic and antitumor activities [7,8,9,10,11], recent attention has been paid to unnatural MeQones from the pharmacological and physiological viewpoints. Indeed, more than 13,000 compounds having the MeQone framework as a partial structure have been reported. However, there is a need to develop new synthesis and functionalization methods for the MeQone framework involving easy experimental manipulations under mild conditions. In particular, it is very important to find a useful scaffold leading to various kinds of compounds, which enables the construction of a new compound libraries for medicinal chemistry.

**Figure 1:**
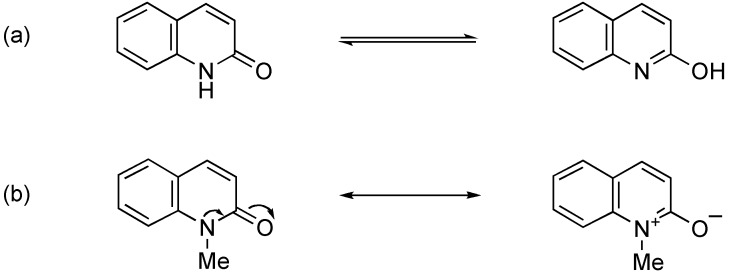
**(a)** Tautomerism of 2-quinolone. **(b)** Resonance structures of 1-methyl-2-quinolone (MeQone).

The quinolone framework is composed of a pyridone moiety and a fused benzene moiety. *N*-Unsubstituted quinolone exhibits aromaticity because of the tautomeric quinolinol structure. Although *N*-substituted quinolones such as MeQone cannot tautomerize to the quinolinol form, they have slight aromaticity due to the contribution of the betaine resonance structure, as illustrated in Figure 1(b). The aromaticity diminishes the reactivity of MeQone itself preventing both direct nucleophilic and electrophilic functionalization. The MeQone ring is often activated by an electron-donating hydroxy group for introduction of a substituent at the adjacent position, and then the hydroxy group is condensed with a newly introduced substituent to form an additional ring fused on the pyridone ring [12,13,14,15,16]. This method is one of the general approaches to natural and unnatural MeQone derivatives. On the other hand, activation of the MeQone framework by an electron-withdrawing group is also possible, however, this strategy is not so familiar yet for researchers in this field. The present review describes the chemistry of nitrated MeQones, especially focusing on 1-methyl-3,6,8-trinitro-2-quinolone (abbreviated as **TNQ** hereafter).

## 2. Preparation of Nitroquinolones

**Scheme 1:**
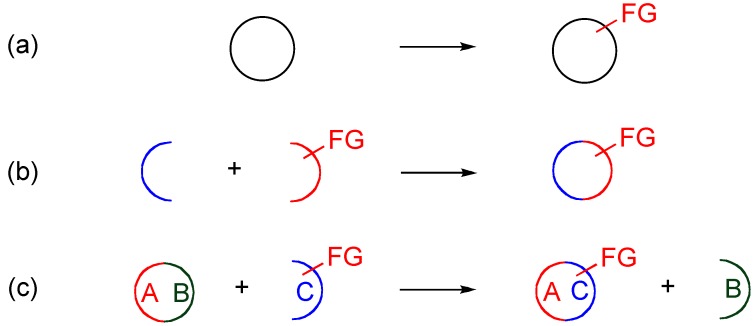
**(a)** Direct functionalization. **(b)** Built-in method. **(c)** Ring transformation.

Preparative methods for functionalized heterocyclic compounds are generally divided to three categories, as illustrated in Scheme 1, namely: (a) direct functionalization of the heterocyclic ring, (b) built-in methods using a functionalized building block, and (c) ring transformations, which are supplementary to each other. These methodologies are also applicable to the preparation of nitroquinolones.

### 2.1. Nitration of the MeQone Framework

**Scheme 2:**
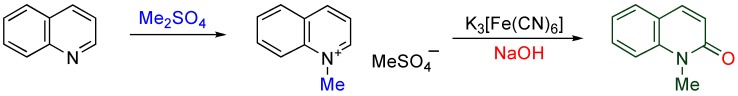
Preparation of MeQone from quinoline.

The starting MeQone is available by chemoselective methylation of 2-quinolone (carbostyril) with chloromethyldimethylsilyl chloride [17]. MeQone is also prepared from quinoline in one-pot by methylation of quinoline with dimethyl sulfate, followed by oxidation using potassium ferricyanide(III) under alkaline conditions (Scheme 2) [18,19].

Nitration of MeQone with fuming nitric acid (d = 1.52) goes to completion and leading to **TNQ** in 90% yield (Table 1). Use of 15 M nitric acid instead of fuming acid at same temperature also affords **TNQ**, although in lower yield. It is possible to obtain the intermediate mono- and di-nitrated MeQones by conducting the nitration at lower temperatures. 1-Methyl-6-nitro-2-quinolone (**6-NQ**) is mainly produced in a good yield at 50 °C, and the dinitrated products 3,6-dinitro-1-methyl-2-quinolone (**3,6-DNQ**) and 6,8-dinitro-1-methyl-2-quinolone (**6,8-DNQ**), are formed at medium temperature [20]. On the basis of these results, the nitro group is introduced in the order of 6- > 3- ≈ 8-positions in which nitration at the 3-position is somewhat easier than at the 8-position.

**Table 1:** Preparation of nitroquinolones by nitration of MeQone. 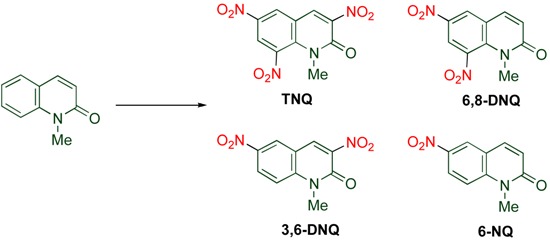

Nitrating Reagent	Temp. / °C	Yield / %			
		TNQ	6,6-DNQ	3,6-DNQ	6-NQ
fuming HNO_3_	120	90	0	0	0
HNO_3_ / H_2_SO_4_	120	63	0	0	0
HNO_3_ / H_2_SO_4_	80	8	29	41	18
HNO_3_ / H_2_SO_4_	70	4	10	26	19
HNO_3_ / H_2_SO_4_	50	0	0	0	72

**Scheme 3:**
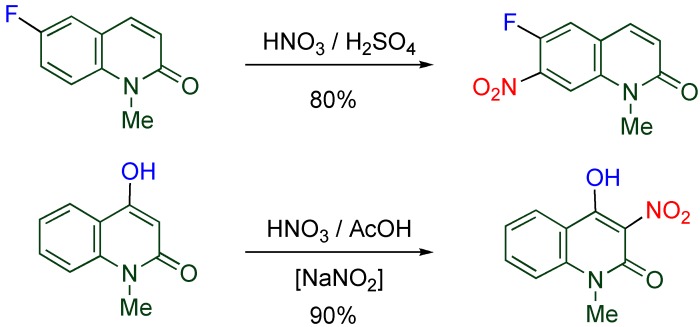
Nitration of substituted MeQones.

In the unsubstituted MeQone, the ring nitrogen at the 1-position serves as an electron-donating group to activate the 3-, the 6- and the 8-positions, however the positional reactivity is changed with an additional substituent (Scheme 3). When the 6-position is blocked with a fluoro group, nitration takes place at the 7-position predominantly [21]. On the other hand, introduction of an electron-donating hydroxy group at the 4-position activates the pyridone moiety, the adjacent 3-position is the most reactive for the nitration in which sodium nitrite is added as an initiator. The resultant vicinal functionalities are useful for the successive construction of a new condensed ring [22,23]. 

### 2.2. Built-in Method Using Ethyl Nitroacetate

**Scheme 4:**
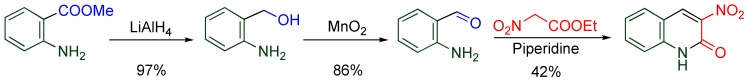
Preparation of nitroquinolones from methyl anthranilate.

Ethyl nitroacetate is a useful building block which constructs a ring having both a nitro and an oxo group. This ester is available from commercial sources or is prepared from nitromethane by a two step reactions [24]. 3-Nitro-2-quinolone is synthesized by condensation of nitroacetate with *o*-formylaniline that is prepared from anthranilic acid ester via *o*-hydroxymethylaniline (Scheme 4) [25]. 

### 2.3. Ring Transformation of Isatoic Anhydride

Ring transformation is also a useful procedure for construction of functionalized heterocyclic compounds which are not easily prepared by alternative methods. Substrates for such reactions are required to have a good leaving group as a partial structure. From the viewpoint of constructing the MeQone framework, isatoic anhydride is the most suitable substrate (Table 2). MeQones having a nitro group on the benzene ring are available by the ring transformation of nitrated isatoic anhydrides with dimethyl malonate accompanying decarboxylation [26,27]. The use of ethyl nitroacetate instead of dimethyl malonate enables the synthesis of 3-nitro-2-quinolone [28,29].

**Table 2:** Ring transformation of isatoic anhydride leading to MeQones.

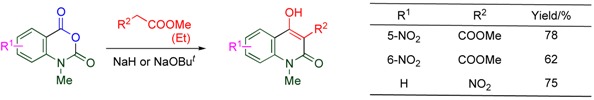

**Scheme 5:**
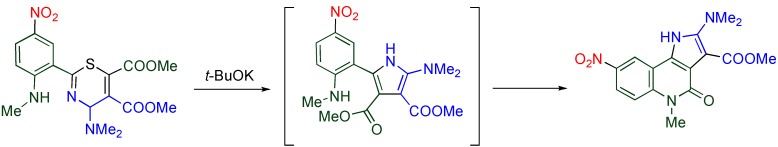
Ring transformation of aminophenyl-1,3-thiazine.

As another example, 2-(2-aminophenyl)-1,3-thiazine derivatives are also usable as a substrate for the ring transformation, which results in the formation of pyrroloquinoline derivatives. In the present reaction, 2-(2-aminophenyl)pyrrole is formed as an intermediate, and the subsequent intramolecular nucleophilic substitution forms the pyridone moiety (Scheme 5) [30].

## 3. Reactivity of Nitroquinolones

### 3.1. Chemical Transformation of a Nitro Group

The nitro group has high potential in organic syntheses as it can be transformed to versatile functionalities. The Nef reaction converts nitroalkanes into ketones directly upon treatment under basic conditions [31,32]. Moreover, reduction of a nitro group leads to an amino group which can be subjected to further chemical transformations affording versatile functionalities via diazonium salts. 

**Scheme 6:**
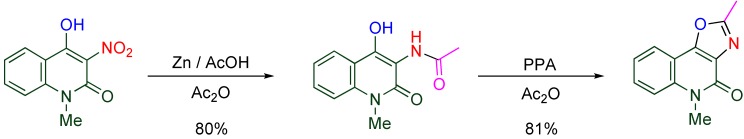
Construction of the [*c*]-fused isoxazole ring.

The vicinal functions of 4-hydroxy-3-nitro-2-quinolone are used for construction of a fused ring on the [*c*]-face. When the nitro group was reduced with zinc in acetic acid in the presence of acetic anhydride, an 3-acetylamino derivative is formed, whose acetyl group protects the sensitive amino group. The subsequent ring closure proceeds by heating in acetic anhydride with polyphosphoric acid at 150 °C to give an isoxazoloquinoline (Scheme 6) [33].

**Scheme 7:**
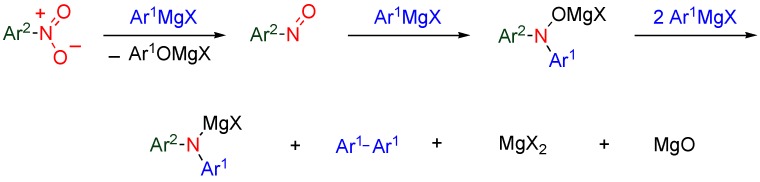
Reaction of nitroarene and aryl Grignard reagent.

A nitro group on the aromatic ring reacts with aryl Grignard reagents to afford diarylamines via hydroxylamine species (Scheme 7). 5,8-Dimethoxy-6-nitroquinolone reacts with aryl Grignards to furnish 6-arylamino derivatives (Table 3). The subsequent treatment of the product with palladium acetate causes successive oxidative coupling and demethylation to afford pyrido[3,2-*b*]carbazolequinones whose intercalating properties are expected to result in anti-tumor activity [34].

**Table 3:** Arylamination of the 6-nitro group followed by conversion to pyridocarbazole.

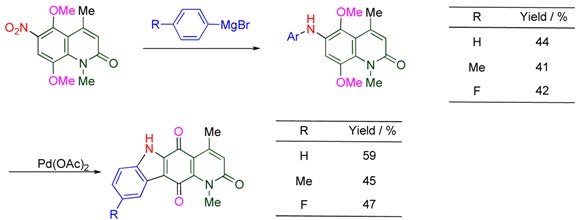

### 3.2. Activation of the Vicinal Position by a Nitro Group

The electron-withdrawing ability of the nitro group corresponds to those of two chloro groups, which strongly activates the attached framework. The vicinal position of the nitro group is especially electron-deficient because of its both electron-withdrawing inductive and resonance effects. When 5,7-dimethoxy-6-nitroquinolone is subjected to reaction with aryl Grignards, arylation occurs at the 5-position, in addition to the arylamination at the 6-position mentioned in the previous section [34]. On the other hand, only substitution at the 5-position is observed in the corresponding reactions with vinyl Grignards (Table 4) [35].

**Table 4:** Substitution with Grignard reagents.

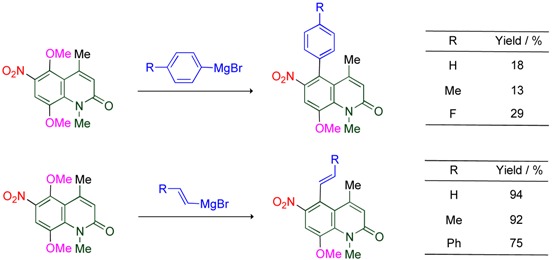

**Scheme 8:**
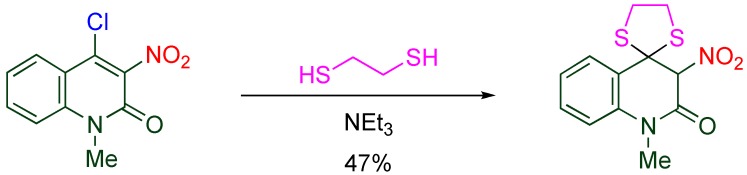
Tandem addition of ethanedithiol leading to a spiro compound.

The 4-position of 4-chloro-3-nitroquinolone is highly active for the nucleophilic substitution giving 4-functionalized 3-nitroquinolones. By this method, functional groups such as azide, amino, fluoro, alkoxy, alkylthio groups, and malonates are introduced at this position (Table 5) [22,36,37]. In addition, intramolecular conjugate addition proceeds to afford spiro thioacetal when ethanedithiol is used as the nucelophile (Scheme 8) [38].

**Table 5:** Nucleophilic substitution of 4-chloro-3-nitro-2-quinolone. 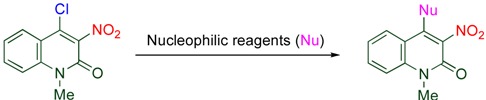

Nucleophilic reagent	Nu	Yield/%		Nucleophilic reagent	Nu	Yield/%
KF + 18-Crown-6	F	95		NH_3_	NH_2_	91
MeONa	MeO	85		PhCH_2_NH_2_	PhCH_2_NH	94
PhOH + K_2_CO_3_	PhO	93		PhNH_2_	PhNH	98
EtSH + NEt_3_	EtS	95		piperidine	piperidino	96
PhSH + pyridine	PhS	96		CH_2_(COOMe)_2_ + K_2_CO_3_	CH(COOMe)_2_	95
NaN_3_	N_3_	95		AcCH_2_COOEt + K_2_CO_3_	AcCHCOOEt	90

**Scheme 9:**
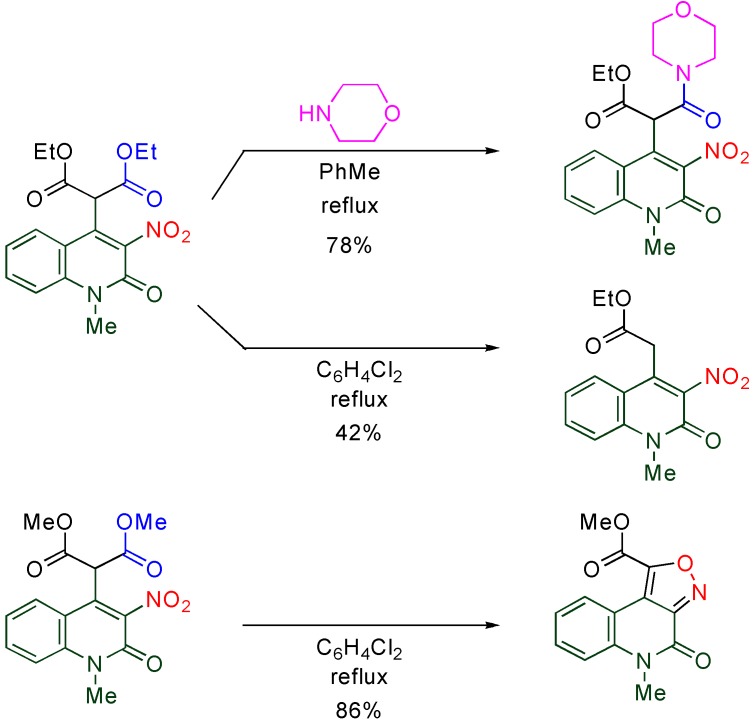
Chemical transformation of malonylquinolones.

Although malonates are not so reactive under usual conditions, reactivity is improved when they are substituted on the MeQone ring. An unsymmetrical amide ester is readily formed upon treatment of quinolylmalonate with morpholine [36]. Interestingly, the reactivity of quinolylmalonate varies with the alkoxy group of the ester function. While dealkoxycarbonylation proceeds in the case of diethyl ester, an isoxazole ring is constructed on the [*c*]-face in the case of the dimethyl ester (Scheme 9) [37]. 

**Scheme 10:**
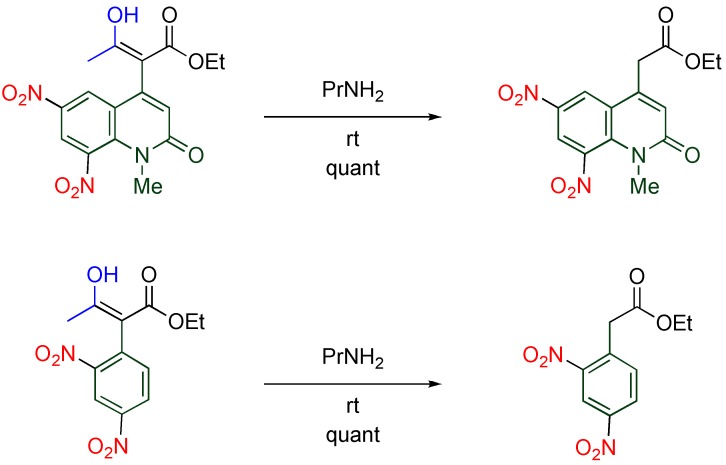
Activation of the acyl group of α-aryl-β-keto esters.

The nitro group at the 3-position seems to activate the diester function at the vicinal position, however, no reasonable explanation has been given for the different reactivity between dimethyl and diethyl esters. Meanwhile, the acyl group of β-keto ester is considerably activated by a quinolyl group at the α-position, [18] and similar activation is achieved by a benzene ring (Scheme 10) [39]. Thus, the bulkiness of the MeQone ring is considered to be an influential factor (See also Section 4.3).

### 3.3. Cycloaddition of Nitroalkene Moiety

**Scheme 11:**
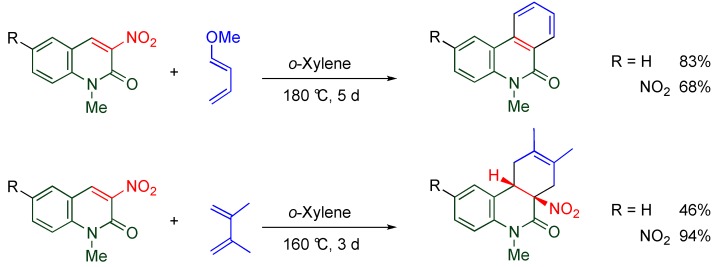
Diels-Alder reactions of 3-nitroquinolones.

MeQones having a nitro group at the 3- or the 4-position serve as nitroalkenes that undergo Diels-Alder reactions with electron-rich dienes leading to benzoquinoline derivatives, in which the constructed ring aromatizes accompanied by elimination of nitrous acid (Scheme 11). Although this methodology enables simultaneous C-C bond formation at the 3- and 4-positions of the MeQone framework, severe reaction conditions must be employed [40,41] To the contrary, trinitroquinolone (**TNQ**) quite easily undergoes cycloaddition under mild conditions as detailed in the next section.

## 4. Unusual Reactivity of 1-Methyl-3,6,8-trinitroquinolone (TNQ)

**Scheme 12:**
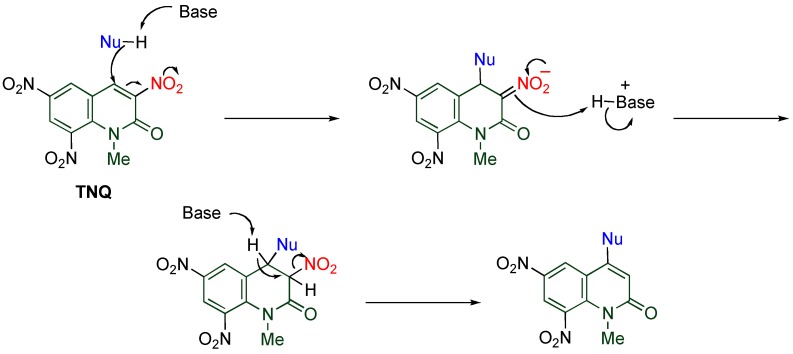
*cine*-Substitution of **TNQ**.

At a glance polynitrated quinolones would appear to be explosive, however, even **TNQ** is quite stable under usual conditions, which enables the storage and the use of nitroquinolones without special care. Despite this stability, **TNQ** exhibits significantly high reactivity, which is unusual compared with other nitroquinolones. Indeed, **TNQ** reacts with versatile nucleophiles to undergo *cine*-substitution, in which the nucleophile substitutes at the 4-position accompanied by elimination of the vicinal nitro group leading to 4-substituted 6,8-dinitro-1-methyl-2-quinolones (Scheme 12). This reaction enables the regioselective functionalization at the 4-position with forming a C-N or a C-C bond. Furthermore, **TNQ** undergoes cycloaddition efficiently under mild conditions to afford polycyclic MeQone derivatives. The high reactivity of **TNQ** is caused by the presence of a nitro group at the 8-position, which is also mentioned in the present section.

### 4.1. Reactions with Amines

The conjugate addition of primary amines readily occurs at the 4-position of **TNQ**, and the resulting adduct is converted to an ammonium salt by another molecule of amine. When the salt is heated, a small amounts of *cine*-substituted product is formed, together with recovery of a large amount of **TNQ**. In this reaction, 3,4-dihydroquinolone is formed under equilibrium at reflux temperature, from which the former product is afforded by deprotonation at the 4-position via **route a**, and the latter product is a result of deprotonation at the 3-position via **route b** (Table 6) [42].

**Table 6:** Reaction of **TNQ** with primary amines.

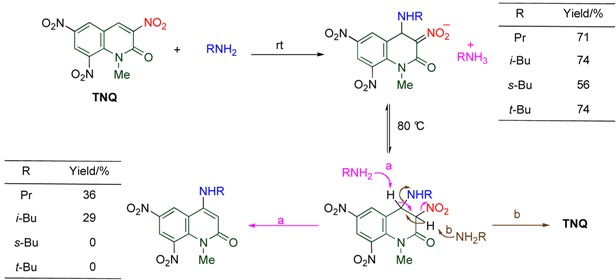

On the other hand, dimerization proceeds in the reaction of **TNQ** with less nucleophilic tertiary amines, by which the dimer connected between the 3- and the 4’-positions is formed. The reaction rate is significantly affected by the length and the number of alkyl chains of the amine (Table 7). Namely, tributylamine causes the reaction faster than tripropylamine and triethylamine, and no reaction proceeds in the cases of trimethylamine and tribenzylamine. In addition, more than two long alkyl chains are necessary for the reaction to occur efficiently [20].

**Table 7:** Dimerization of **TNQ** depending on amine structure. 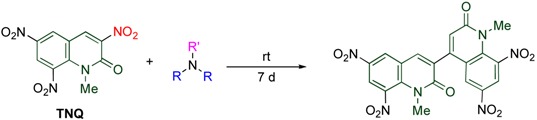

R	R’	Yield/%
Bu	Bu	93
Pr	Pr	76
Et	Et	34
Bu	Me	79
Me	Bu	18

**Scheme 13:**
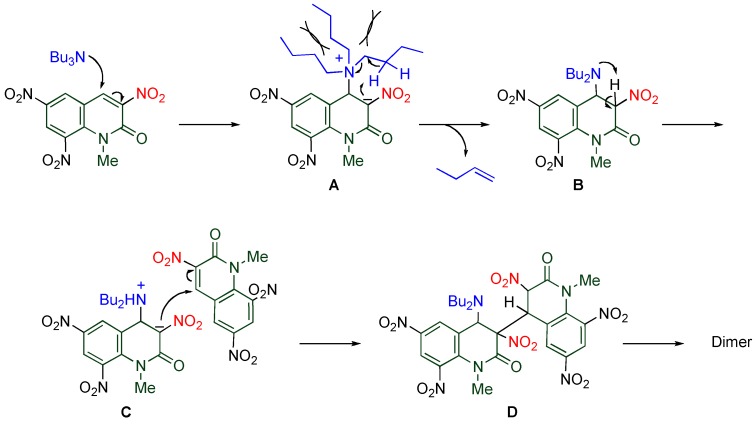
A plausible mechanism for the dimerization of **TNQ**.

A plausible mechanism is illustrated in Scheme 13. The reaction is initiated by addition of tributylamine to the electron-deficient 4-position of **TNQ** yielding zwitterion **A**, from which β-elimination releasing 1-butene proceeds to afford dihydroquinolone **B**. The successive proton transfer from the 3-position to the adjacent dibutylamino group leads to zwitterion **C** which then reacts with another **TNQ **to cause *cine*-substitution, giving the dimer via dihydroquinolone adduct **D**. The key step of the present reaction is the intramolecular prototropy of zwitterion **A **accompanying elimination of an alkyl chain as an alkene. Since trimethylamine and tribenzylamine have no β-hydrogens, only elimination of tertiary amine from the adduct proceeds to give **TNQ** again. In the case of alkyldimethylamines, the alkyl chain avoids steric repulsion with the adjacent nitro group, and the suitable conformation for β-elimination barely occurs. On the other hand, one of the alkyl chains surely locates nearby the 3-nitro group when the amino group has more than two alkyl groups. Hence, tertiary amines should have proper steric bulk for a smooth reaction [20].

### 4.2. Cine-Substitution by 1,3-Dicarbonyl Compounds

The *cine*-substitution of **TNQ** by C-H acids is a useful protocol for regioselectively forming a C-C bond at the 4-position leading to versatile skeletons. 1,3-Dicarbonyl compounds such as β-diketones, β-keto esters and β-diesters easily react with **TNQ** in the presence of triethylamine at room temperature to afford 4-functionalized 6,8-dinitro-2-quinolones (Table 8) [18].

**Table 8:** Reactions of nitroquinolones with 1,3-dicarbonyl compounds. 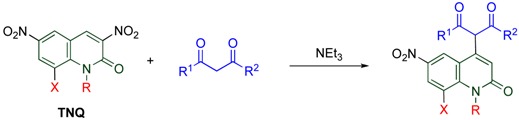

X	R		R^1^	R^2^	Yield/%
NO_2_	Me	**(TNQ)**	Me	Me	88
NO_2_	Me	**(TNQ)**	-(CH_2_)_3_-		60
NO_2_	Me	**(TNQ)**	Me	OEt	93
NO_2_	Me	**(TNQ)**	CH_2_COOEt	OEt	26
NO_2_	Me	**(TNQ)**	OEt	OEt	99
NO_2_	H	**(TNQ-H)**	Me	Me	0
H	Me	**(3,6-DNQ)**	Me	Me	0

On the other hand, neither demethylated trinitroquinolone (**TNQ-H**) nor 3,6-dinitroquinolone (**3,6-DNQ**) causes *cine*-substitution, even at elevated temperature. These facts suggest that both 1-methyl and 8-nitro groups are necessary for the high reactivity of **TNQ** [43].

**Figure 2:**
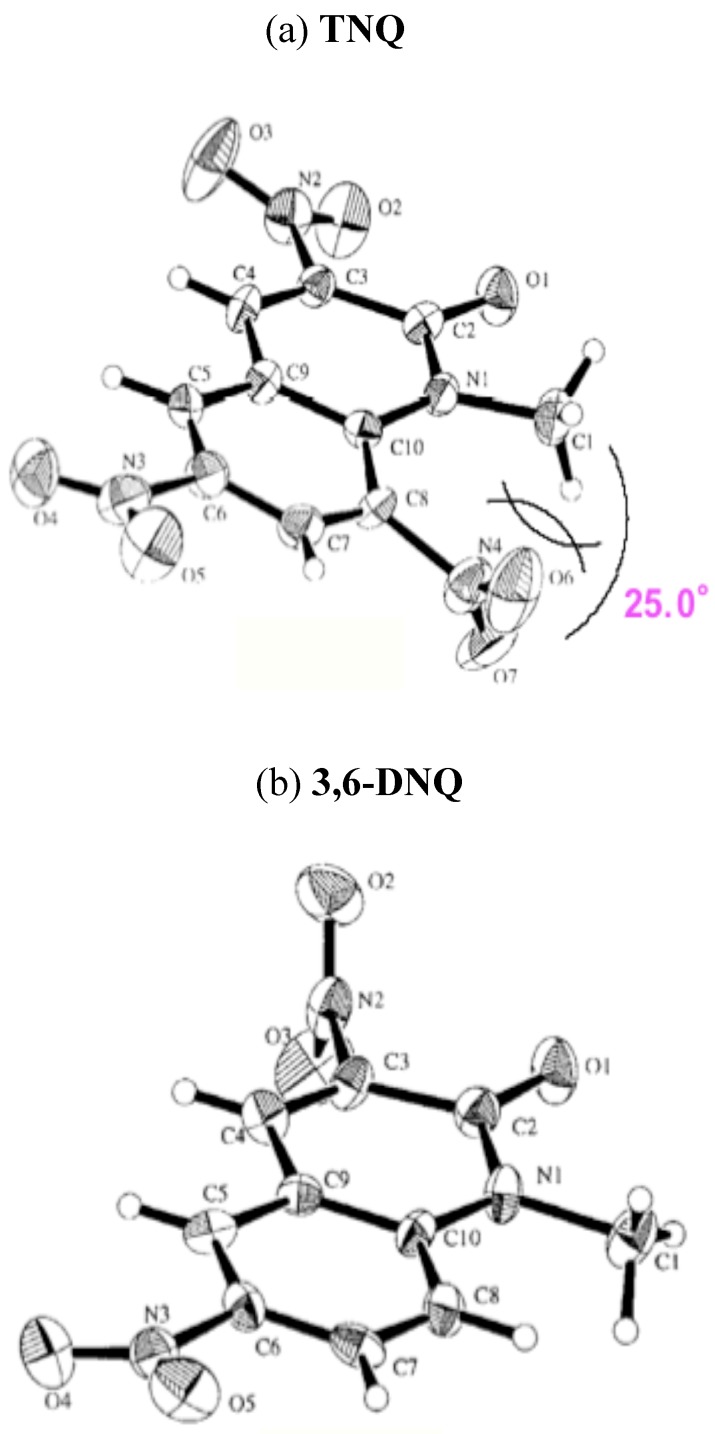
**(a)** An ORTEP view of **TNQ**. **(b)** An ORTEP view of **3,6-DNQ**. (30% probability ellipsoids).

MOPAC (PM3) molecular orbital calculations for **3,6-DNQ**, and **TNQ-H **reveal that both the benzene and pyridone rings present are almost coplanar. In contrast, the 8-nitro group of **TNQ-Me **has no coplanarity with the quinolone ring, which turns through 67.7°. Furthermore, the 2-quinolone ring is torsionally strained by the steric compression of the 1-methyl and the 8-nitro groups. The dihedralangle between C8-NO2 bond and N1-Me one is 30.0° (Table 9). Indeed, X-ray analyses for **3,6-DNQ** and **TNQ** agree well with the calculated results; the 8-NO2 group of **TNQ **was turned through 55.8° and the dihedral angle is 25.0° (Figure 2). From the agreed results of X-ray analyses to calculated ones for **TNQ **and **3,6-DNQ**, the actual dihedral angle between the pyridone ring and the benzene ring of **TNQ-H **is also presumed to be a small [43]. 

**Table 9:** Dihedral angles between 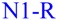 and 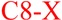 bonds. 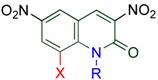

	R		Calculated	Actual
NO_2_	Me	**TNQ**	30.0°	25.0°
H	Me	**3,6-DNQ**	0.7°	0.9°
NO_2_	H	**TNQ-H**	0.04°	−−

The 8-nitro group cannot diminish the electron density of the reaction site (the 4-position) because of the large distance and the fact its torsion interferes with the conjugation, thus, the MeQone framework is activated sterically rather than electronically. Namely, the nitro group significantly distorts the quinolone ring by steric repulsion with the 1-methyl group. As a result, the pyridone moiety of the MeQone cannot be coplanar with the benzene ring, which prevents the π-orbitals from overlapping effectively. Consequently, the pyridone moiety exhibits nitroalkene property rather than aromaticity, and which should be a major reason for the extremely high reactivity of **TNQ**.

### 4.3. Chemical Transformation of α-quinolyl-β-diketones and β-keto esters

**Scheme 14:**
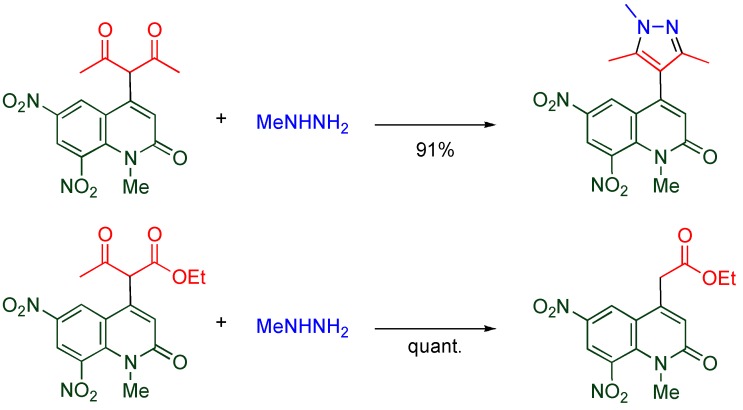
Reaction of α-quinolylated β-diketone and β-keto ester with methylhydrazine.

α-Quinolyl-β-diketone and α-quinolyl-β-keto esters exhibit different reactivity. While the β-diketone is converted to a pyrazole upon treatment with methylhydrazine at room temperature, deacylation proceeds under the same conditions in the case of β-keto esters (Scheme 14). However, it is not necessary to employ the quinolyl group for activating the acyl group of β-keto ester because introduction of a phenyl group results in the similar activation [39]. Thus, the activation of an acyl group is not chemistry specific to MeQones.

### 4.4. Cine-Substitution by Enamines, Ketones, and Nitroalkanes

The *cine*-substitution similarly proceeds when other carbon nucleophiles such as enamines and ketones are employed [44]. In the reaction of **TNQ** with enamines, morpholinium salts of 3,4-dihydro-quinolones are formed, in which intermediate iminium ions are hydrolyzed and deprotonation at the 3-position by the liberated morpholine proceeds (Table 10).

**Table 10:** Reaction of **TNQ** with enamines. 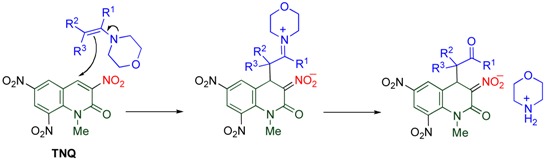

R^1^	R^2^	R^3^	Yield/%
Ph	Me	H	43
Ph	Ph	H	98
-(CH_2_)_3_-		H	40
H	Me	Me	98

**Table 11:** Acylmethylation of the MeQone framework. 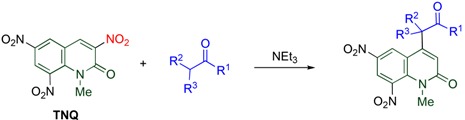

R^1^	R^2^	R^3^	Yield/%
Ph	H	H	83
Ph	Me	H	77
Ph	Ph	H	69
Ph	Me	Me	0
-(CH_2_)_4_-		H	82
Me	H	H	83
Et	Me	H	18
H	Me	Me	41
2-Pyridyl	H	H	74
2-Furyl	H	H	45

Acylmethylation of the MeQone framework is also performed directly by conducting reactions of **TNQ** with ketones in the presence of triethylamine. This reaction is applicable to aliphatic, alicyclic, aromatic, and heteroaromatic ketones (Table 11) [44]. Since the acylmethyl group is expected to serve as scaffold for further chemical transformations, this method would be useful for construction of a new libraries of compounds having a MeQone framework.

**Table 12:** Nitroalkylation of the MeQone framework.

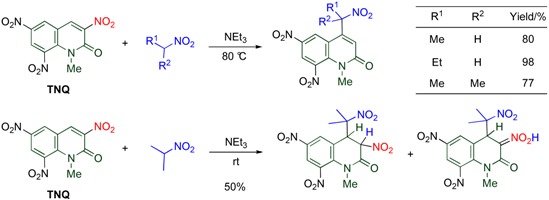

Nitroalkylation is also performed upon treatment of **TNQ** with nitroalkane as a C-H acid (Table 12) [45]. While primary nitroalkanes undergo the *cine*-substitution efficiently, secondary nitroalkanes are less reactive, requiring longer reaction time,. A tautomeric mixture of 3,4-dihydroquinolones is obtained when the reaction is conducted at room temperature.

### 4.5. Cine-Substitution by Phenoxides

Direct arylation of MeQone is one of the more useful modifications from a viewpoint of further functionalization, however, it is quite difficult because the accompanying destruction of the aromaticity of both quinolone and benzene rings. This difficulty is overcome when a combination of electrophilic **TNQ** and nucleophilic phenoxide ions is employed [46].

**Scheme 15:**
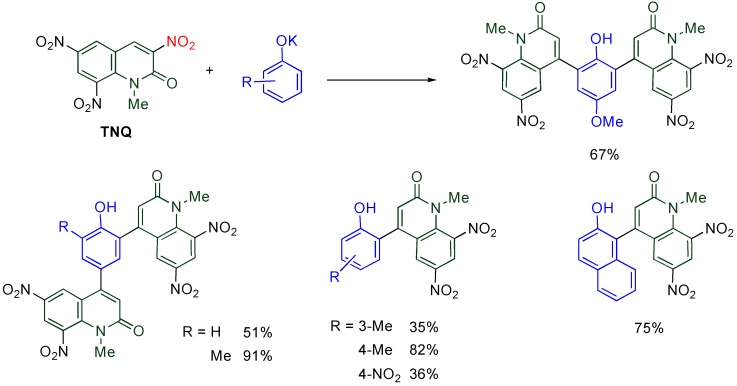
Arylation of the MeQone framework.

While phenol, 2-methylphenol, and 4-methoxyphenol undergo double substitution to afford bis(quinolyl)phenols, bulky or electron-poor phenoxides give monoquinolylphenols as a sole product (Scheme 15). In the reaction of **TNQ** with 2-methylphenoxide, a single substituted product is not detected, even when the molar ratio of reagents is changed from 1/1 to 2/1. These experimental facts indicate that the second substitution proceeds much faster than the first one and half of the amount of phenoxide was consumed as the base.

**Scheme 16:**
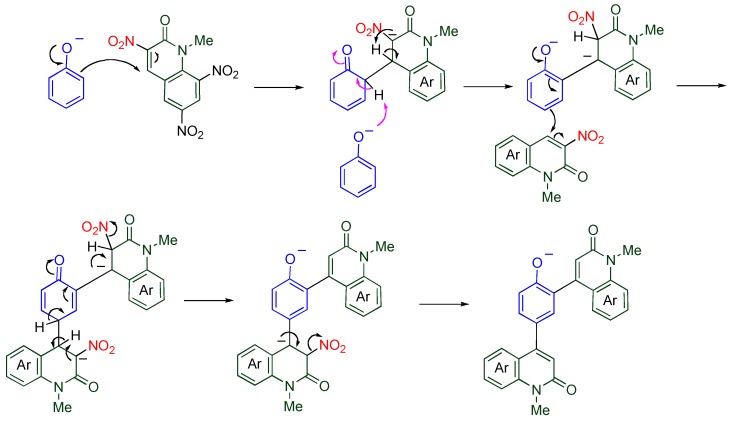
A plausible mechanism for the present arylation

A plausible mechanism is illustrated in Scheme 16. The phenoxide adds to the 4-position of **TNQ**, and another phenoxide assists aromatization of the benzene ring. In the quinolone ring, the proton transfer also occurs from the 4-position to the 3-position. Since the resultant dianionic phenoxide is more reactive than the unsubstituted phenoxide, the second substitution proceeds much faster than the first one. The final product is formed by aromatization of the quinolone ring with loss of nitrite ion [46]. From the standpoint of the benzene ring, the present reaction can be regarded as an electrophilic arylation, which is well-known to be quite difficult, because MeQone is also an aromatic compound.

### 4.6. Cycloaddition of TNQ

**Scheme 17:**
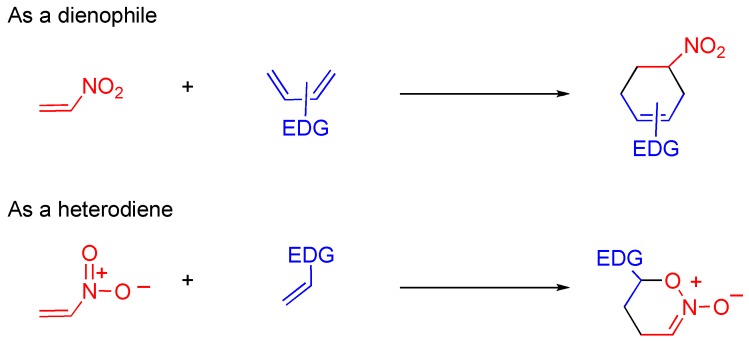
Dual reactivity of nitroalkene in the cycloaddition.

**Scheme 18:**
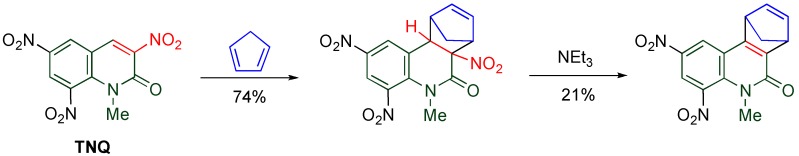
Diels-Alder reaction of **TNQ** with cyclopentadiene.

Nitroalkenes are often used for cycloaddition in which they show dual reactivity as dienophile and heterodiene, under different conditions (Scheme 17) [47,48,49]. As mentioned in Section 4.2, the pyridone moiety reveals nitroalkene properties rather than aromaticity, which means that the C3-C4 moiety of **TNQ** causes cycloaddition under mild conditions while Diels-Alder reactions of 3-nitroquinlone and 3,6-dinitroquinolone with electron-rich dienes require quite severe conditions; *i.e.*, at 180 °C for 5 days in *o*-xylene (Scheme 11) [40,41]. Indeed, efficient cycloaddition proceeds leading to tetracyclic compounds when **TNQ** is allowed to react with cyclopentadiene at 80 °C (Scheme 18) [50]. The cycloadduct aromatizes with elimination of nitrous acid upon treatment with triethylamine.

**Scheme 19:**
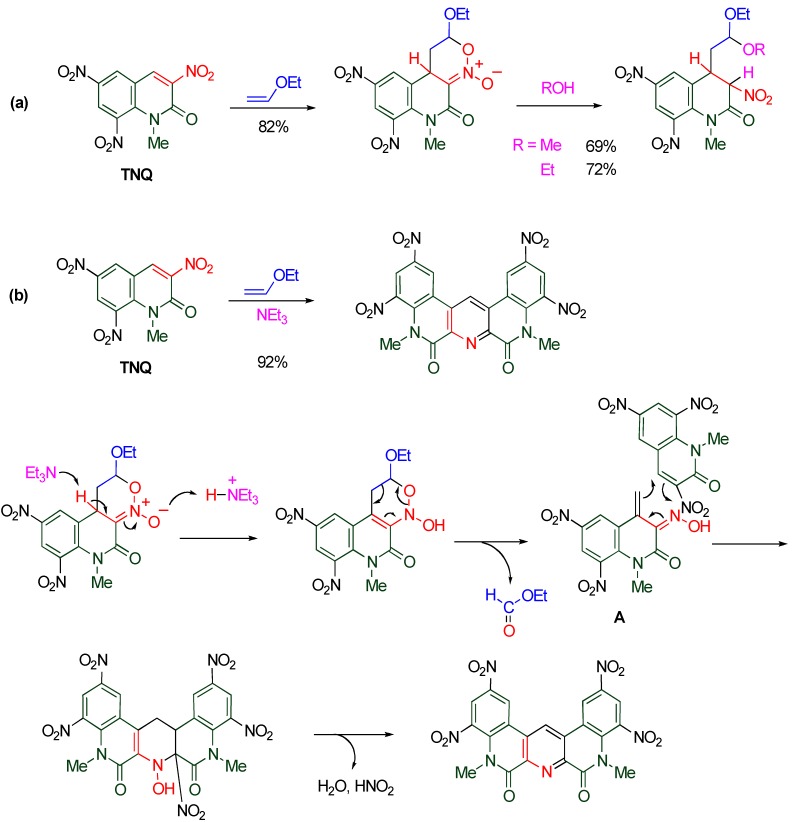
**(a)** Cycloaddition with ethoxyethene in the absence of NEt_3_. **(b)** Cycloaddition with ethoxyethene in the presence of NEt_3_.

The nitroalkene moiety of **TNQ** also serves as a heterodiene in the reaction with ethoxyethene to construct a fused oxazine ring. The cycloadduct is easily converted to an acetal by the ring opening reaction upon heating in alcohol. On the other hand, a quinolino[3,4-*b*][1,9]diazaphenanthrene derivative is formed when the same substrates are treated in the presence of triethylamine (Scheme 19) [51]. 

**Scheme 20:**
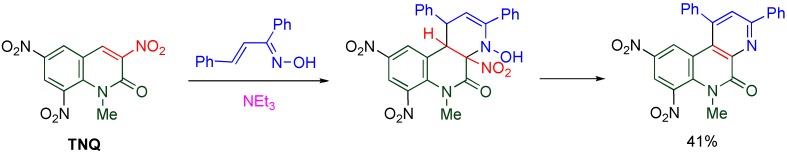
Cycloaddition of **TNQ** with α,β-unsaturated oxime.

This reaction is initiated by cycloaddition of **TNQ** with ethoxyethene affording a cyclic nitronate. Triethylamine accelerates the prototropy from the pyridine ring to the oxygen atom of the nitronate, and then, a retro Diels-Alder reaction occurs to give the α,β-unsaturated oxime **A **with a loss of ethyl formate. The cycloaddition of intermediate **A** with another molecule of **TNQ **constructs a new pyridine ring, and the subsequent aromatization furnishes the polycyclic product together with elimination of nitrous acid and water. In the present process, the former oxime **A **behaves as an electron-rich heterodiene and the latter **TNQ **behaves as an electron-poor dienophile [51]. This mechanism is supported by the experimental fact that polycyclic diazaphenanthrene is isolated in a moderate yield as a result of cycloaddition of **TNQ** with α,β-unsaturated oxime as the electron-rich heterodiene (Scheme 20).

In general, dual reactivity is not observed at the same time except for a single example [52] which yields both products under same conditions, however, nitro substituted bicyclo[2.2.2]octane is formed from cyclic nitronate via [3,3]-sigmatropic rearrangement. Thus, this is the first example showing dual reactivity in the same reaction system under mild conditions.

## 5. Conclusions

A variety of unnatural MeQone derivatives are easily prepared from nitroquinolones, in which the nitro groups in the MeQone framework are efficiently activated to undergo the chemical transformations. Especially, the nitro group at the 8-position activates the MeQone framework sterically in the case of **TNQ** exhibiting unusual reactivity. When **TNQ** is treated with nucleophilic reagents, *cine*-substitution proceeds to form a C-C bond at the 4-position regioselectively. Moreover, polycyclic compounds having the MeQone framework as a partial structure are readily prepared by cycloadditions using the nitroalkene properties of **TNQ**. Nitroquinolones are useful scaffolds for constructing a library of MeQone derivatives, and are expected to exhibit new reactivity in the future.
